# The Impact of Sedentary Behavior and Self-Rated Health on Cardiovascular Disease and Cancer among South Korean Elderly Persons Using the Korea National Health and Nutrition Examination Survey (KNHANES) 2014–2018 Data

**DOI:** 10.3390/ijerph18147426

**Published:** 2021-07-12

**Authors:** Soojin Park, Jin Young Nam

**Affiliations:** Department of Healthcare Management, Eulji University, Sungnam-si 13135, Korea; sj_eu@naver.com

**Keywords:** sedentary behavior, self-rated health, cardiovascular disease, cancer, elderly

## Abstract

Cardiovascular disease and cancer have increased the risk of mortality and morbidity in elderly persons worldwide. The aim of this study was to investigate the association of sedentary behavior and self-rated health with cardiovascular disease or cancer in elderly people. The data of 6785 elderly persons aged above 65 years from the Korea National Health and Nutrition Examination Survey 2014–2018 were examined. Binary logistic regression analyses assessed the association of sedentary behavior, self-rated health, and other risk factors with cardiovascular disease or cancer. Prolonged sedentary behavior in elderly people was associated with a high risk for cardiovascular disease (odds ratio (OR): 1.28, 95% confidence interval (CI): 1.08–1.52). There was a high risk for cardiovascular disease (OR: 2.36, 95% CI: 1.85–3.01) or cancer (OR: 1.48, 95% CI: 1.17–1.88) in elderly people who had poor self-rated health. This study identified the association between prolonged sedentary behavior and cardiovascular disease, and between poor self-rated health and cancer. Since prolonged sedentary behavior is related to cardiovascular disease, efforts are needed to reduce sedentary behavior hours and maintain good self-rated health.

## 1. Introduction

The growing elderly population has led to the introduction of the concept of “healthy aging” and life support. Cardiovascular disease (CVD) and cancer have increased the risk of mortality and morbidity in elderly persons aged 65 years and above worldwide [1]. However, adopting healthy lifestyles can help in the prevention of these diseases [2]. 

Sedentary behavior (SB) is an important contributing factor to chronic diseases and cancer [3]. Long sedentary or lying down time is associated with health deterioration and unhealthy aging [4]. The World Health Organization (WHO) recommends 150 min of medium- to high-intensity activities in bouts of at least 10 min every week [5]. A high level of sedentarism leads to chronic diseases like CVDs, which are the leading cause of mortality, especially in elderly people [3,6]. However, it has been observed that elderly people spend about 60% (8.5–9 h) of their waking hours in sedentary behaviors because of their physical and social constraints [7].

With the increasing elderly population worldwide, the prevalence of chronic diseases is also increasing, and their self-rated health (SRH) is getting worse [8]. SRH varies from individual to individual, such that a person may feel unhealthy even in the absence of any disease, while some persons with several or many diseases may consider themselves as healthy [9]. A person with a good SRH is highly satisfied with their life. SRH is an economical and convenient predictor of chronic diseases, mortality, and functional loss [10]. SRH is important in evaluating the quality of life and disease in elderly persons who undergo physical, mental, and social changes [10].

SB is one of the factors that affect SRH; studies from different countries have found an association between SB and SRH [4,11]. The physical activity, lifestyle, and health of the elderly in South Korea have been of significant interest to researchers [12]. The proportion of the elderly in the general population and the proportion of national and social policies on the health of the elderly are increasing [13]. To provide sufficient supporting evidence for developing policies concerning the elderly, this study aimed to identify the association of sedentary behavior and SRH with CVD or cancer in the elderly.

## 2. Materials and Methods

### 2.1. Study Participants and Database Information

The Korea National Health and Nutrition Examination Survey (KNHANES) is an annual survey program that was initiated in 1998 by the Korea Centers for Disease Control and Prevention (KCDC) to monitor the lifestyle of people. KNHANES collects data on smoking, physical activity, diseases or medical conditions, and the nutritional status of the general population of South Korea. This study utilized the data of the KNHANES 2014–2018 participants who were above 65 years old. Among 39,199 participants, we excluded participants aged <65 years (*n* = 31,109) and participants with insufficient data on sedentary behavior (*n* = 1304) or SRH (*n* = 836). As a result, 6785 participants were included in this study. This study was approved by the KCDC Institutional Review Board (IRB; No. 2013-12EXP-03-5C for 2014, 2018-01-03-P-A for 2018). The KNHANES was implemented without an IRB review in 2015–2017, according to the Bioethics Act and Enforcement Rules.

### 2.2. Sedentary Behavior 

SB is defined as “activities with an energy expenditure of ≤1.5 METs while sitting or lying down at work or home, traveling, or reclining with peers, excluding sleep time behavior” [14]. In this study, SB was assessed using the following question: “How much time do you spend sitting or lying down a day?”. The participants’ responses were in hours. The responses were categorized into 0–6 h/day and ≥7 h/day [15].

### 2.3. Self-Rated Health 

SRH status was measured using the following question: “How do you rate your overall health condition?”. The participants were asked to rate their health as “very good”, “good”, “fair”, “poor”, or “very poor”. The SRH ratings were categorized as good (very good or good) or poor (fair, poor, or very poor). 

### 2.4. Cardiovascular Disease and Cancer 

The outcome variable was the prevalence of CVD and cancer. CVD was defined as stroke, myocardial infarction, and angina. The types of cancers included stomach, liver, colon, breast, cervix, lung, thyroid, and other cancers. The presence of a CVD or cancer was assessed by asking the participants whether the diseases were diagnosed by a physician and whether they were receiving curative or preventive medication for the disease.

### 2.5. Covariates

The covariates that were assessed for socioeconomic status (SES) included sex, age (65–75 years: young-old, and ≥76 years: old-old), education level (middle school or lower/high school or higher), household income (discretized based on quintiles), marital status ( “yes” (married) or “no” (unmarried, divorced, widowed, or separated)), physical activity, waist circumference, current smoking, high-risk alcohol consumption, and chronic diseases (diabetes mellitus, hypertension, dyslipidemia). Physical activity level was evaluated using the Global Physical Activity Questionnaire (GPAQ) Korean version [16], which was developed based on WHO’s physical activity guidelines [17]. A waist circumference of ≥90 for men and ≥80 cm for women was categorized as obese, according to the Asia-Pacific regional guidelines of WHO [18]. Current smokers were those who had smoked five packs (≥100 cigarettes) in their lifetime. High-risk alcohol consumption was defined as drinking ≥7 drinks/week for men and ≥5 drinks/week for women twice or more per week. 

### 2.6. Statistical Analysis

The participants’ characteristics were analyzed using the chi-square test. A binary logistic regression analysis was performed to analyze the association between SB, SRH, and risk factors and CVD or cancer; the results are expressed as odds ratios (ORs) and 95% confidence intervals (CIs). The combined effect of SB and SRH on CVD and cancer, stratified by sex, was also examined in a binary logistic regression analysis after adjustment for age, household income, education level, marital status, physical activity, current smoking, high-risk alcohol consumption, waist circumference, diabetes mellitus, hypertension, and dyslipidemia. A *p*-value of < 0.05 was defined as statistically significant. All analyses were performed using SAS version 9.4 (SAS, Inc., Cary, NC, USA). 

## 3. Results

The characteristics of the total study population are shown in Table 1. Of the 6785 participants, 914 (13.5%) had CVD and 663 (9.8%) had cancer. Of the 4156 participants with SB hours above 7 h, 609 (14.7%) had CVD (*p* < 0.01) and 420 (10.1%) had cancer. A total of 818 (23.0%) participants with CVD had a poor SRH and 552 (10.3%) participants with cancer had a poor SRH (*p* < 0.001). Of those with CVD, 419 (15.2%) participants were obese, 268 (17.9%) had diabetes mellitus, 660 (15.5%) had hypertension, and 362 (15.1%) had dyslipidemia. Of those with cancer, 440 (8.9%) individuals had a low educational level, 408 (10.5%) were obese, and 389 (9.2%) had hypertension (*p* < 0.05) (Table 1).

Table 2 shows the association of SB and SRH with CVD or cancer. CVD was associated with SB hours above 7 h (OR: 1.28, 95% CI: 1.08–1.51), poor SRH (OR: 2.36, 95% CI: 1.85–3.01), and hypertension (OR: 1.48, 95% CI: 1.21–1.73), when compared to the reference group. Cancer was associated with the male sex (OR: 1.36, 95% CI: 1.10–1.69), poor SRH (OR: 1.48, 95% CI: 1.17–1.88), and current smoking status (OR: 0.63, 95% CI: 0.45–0.90), when compared to the reference group (Table 2). 

**Figure 1:**
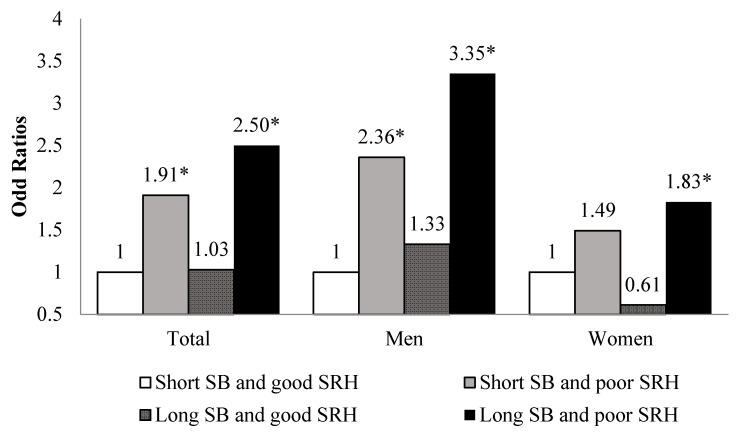
Association of sedentary behavior and self-rated health with cardiovascular disease by sex. Adjusted for age, household income, education level, marital status, physical activity, current smoking, high-risk alcohol consumption, waist circumference, diabetes mellitus, hypertension, dyslipidemia SB, Sedentary behavior; SRH, Self-rated health * p < 0.05.

Figure 1 shows the association of SB and SRH with CVD prevalence by sex. Participants with long SB hours and a poor SRH had a higher risk for CVD than those with short SB hours and a good SRH (OR: 2.50, 95% CI: 1.74–3.58). Participants with long SB hours and a poor SRH had a significantly higher risk for CVD, when stratified by men (OR: 3.35, 95% CI: 2.10–5.33) and women (OR: 1.83, 95% CI: 1.04–3.24), than those who had short SB hours and a good SRH (Figure 1; Supplementary Table S1). 

**Figure 2:**
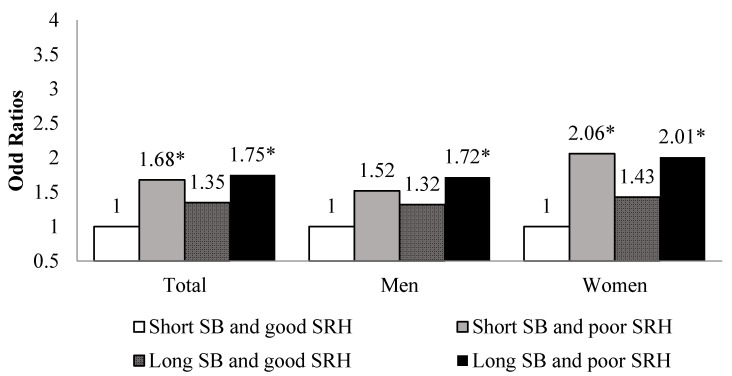
Association of sedentary behavior and self-rated health with cancer by sex. Adjusted for age, household income, education level, marital status, physical activity, current smoking, high-risk alcohol consumption, waist circumference, diabetes mellitus, hypertension, dyslipidemia SB, Sedentary behavior; SRH, Self-rated health * *p* < 0.05.

Figure 2 shows the association of SB and SRH with the prevalence of cancer by sex. Participants with short SB hours and a poor SRH had a higher risk for cancer than participants with short SB hours and a good SRH (OR: 1.68, 95% CI: 1.15–2.46). Among elderly women, participants with long SB hours and a poor SRH had a significantly higher risk for cancer than participants with short SB hours and a good SRH (OR: 2.01, 95% CI: 1.06–3.81) (Figure 2; Supplementary Table S2).

## 4. Discussion

In this study, the association of SB and SRH with CVD and cancer in Korean elderly persons aged 65 or above using the KNHANES data was examined. CVD was associated with SB and SRH, while cancer was associated with self-rated health. These results suggest that older adults need to reduce SB hours to prevent CVD and strive to maintain a good SRH. Previous cross-sectional studies of elderly English and Latin American adults found that elderly people with long SB hours seemed to have a poor SRH [11,19]. Moreover, this is consistent with the results of a previous study in which the CVD risk increased in individuals with more than seven hours of SB [15]. In this study, the risk for CVD was higher among men than women, and higher among the elderly aged 75 years and above than the younger older adults. CVD was also associated with waist circumference, diabetes mellitus, hypertension, and dyslipidemia. Chronic diseases (e.g., diabetes mellitus, hypertension, dyslipidemia) are well-known risk factors for CVD, and emphasis is placed on their management worldwide to improve their outcomes [20,21]. Reducing SB is essential for the management of CVD and chronic diseases in older adults, especially hypertension, diabetes, and dyslipidemia, at a personal and national level [20,22]. Elderly people need to spend less time performing sedentary activities, such as watching television or lying down, and instead spend time with friends in activities such as dancing, jogging, or aerobics.

The risk for cancer was higher among the older adults with a poor SRH than those with a good SRH. Men had a higher risk for cancer than women, which was consistent with the results of a previous study. Ryou et al. reported that elderly men with a poor SHR had a higher risk for cancer than elderly women [23]. In this study, reverse causation was observed between people who smoke cigarettes and cancer (OR: 0.63, 95% CI: 0.45–0.90). Even though the risk for cancer is higher among people with experience in smoking than those without, previous studies have suggested that it is regardless of the type of cigarette [24]. According to a previous study, elderly people with cancer put a lot of effort into reducing other risk factors and preventing associated diseases to improve their overall health [25]. Thus, we consider this to be the reason why there was reverse causation between smokers and cancer.

The associations of CVD and cancer with SB and SRH were analyzed by sex, respectively. Older adults with short SB hours and a poor SRH had a higher risk for CVD than the elderly with short SB hours and a good SRH. The risk for CVD was 2.36 times higher for men and 1.49 times higher for women, but the difference was not significant. In addition, the elderly with prolonged SB and a poor SRH had a higher risk for CVD in both sexes than the reference group. Another cross-sectional study that included 7660 participants from Britain reported that the type of SB varies with the domain. For example, television viewing was associated with a higher risk for obesity and CVD than working while sitting; therefore, the risk for CVD also varies with the purpose for SB [26]. Moreover, what is considered “health” is subjective and varies with individuals; SRH varies with age and sex [23,27]. Although the exact cause of this finding could not be determined because this study was a cross-sectional survey, several studies have advocated that SRH is a valid predictor of CVD, cancer, and general health status [23,28]. Furthermore, people with a good SRH are actually positive about life and less vulnerable to diseases due to psychological factors [27]. Previous studies have confirmed that SB and SRH are related to CVD [29], and if SB continues for a long time, SRH also deteriorates [10,11].

Older adults with short SB hours and a poor SRH had a higher risk for cancer than the elderly with short SB hours and a good SRH. Women had a 2.06-fold higher risk for cancer; this risk was not significant among men. All elderly women with a poor SRH, irrespective of whether they have long or short SB hours have a higher risk for cancer, and the indicators of SRH are important risk factors for disease [23]. Some studies have shown that there is a risk for cancer when SRH is fair or poor, and doctors should pay attention to patients’ SRH [30]. Previous studies have observed SB as an important factor that can influence the SRH status of the elderly when compared to other age groups [4,11]. SB and SRH were related to social and psychological mechanisms; therefore, an increase in activity hours and a reduction of SB hours can improve SRH [4]. In particular, according to another study wherein elderly women had long SB hours or less physical activity than elderly men [31], greater attention should be paid to elderly women during health promotion activities because they have relatively fewer opportunities to exercise due to their muscular strength or household activities than older men. 

This study has some limitations that should be considered. The data in this study were collected using a self-administered questionnaire; therefore, reporting errors or unsatisfactory answers are likely. However, the questionnaire used in the KNHANES was a verified survey tool, and the missing data of outcomes and main variables were deleted. The KNHANES was a cross-sectional survey. Therefore, through this study, it is impossible to establish a causal relationship of SB and SRH with CVD or cancer. In this study, the exact mechanism of the relationship between variables when combined (such as SB and SRH with cancer) is contrary to previous studies that showed a significant association of SB and SRH with cancer [6,32]; this lack of association might be due to the differences in the cancer type and the difference in SB domains. Further research is necessary to study the relationships that may have been limited by the cancer type, age, and SB type (e.g., television, working).

However, this study was able to find the association of SB and SRH with CVD or cancer using a structured questionnaire survey that was conducted in Korea; CVD and cancer are the major causes of mortality in elderly Koreans. The results of this study can be used as evidence in the development of public health policies for elderly people. 

## 5. Conclusions

This study examined the association of sedentary behavior and self-rated health with CVD or cancer among the elderly in Korea. This study suggests that there is a need to reduce elderly people’s SB hours; that is, elderly people have to avoid spending most of their time sitting or lying down because reducing SB hours can positively improve SRH. Therefore, future research is necessary to investigate the underlying reasons for long sitting and lying down time in the elderly. Additionally, policymakers need to focus more on education and health promotion among elderly adults for CVD and cancer prevention and the promotion of healthy aging.

## Figures and Tables

**Table 1:** General characteristics of the study population according to cardiovascular disease and cancer.

Variables		Cardiovascular Disease (CVD)			Cancer		
	Total	Yes (*n* = 914)	No (*n* = 5871)	*p*-Value	Yes (*n* = 663)	No (*n* = 6122)	*p*-Value
		*N* (%)	*N* (%)		*N* (%)	*N* (%)	
Sedentary behavior (hours)							
0–6	2629	305 (11.6)	2324 (88.4)	0.0003	243 (9.2)	2386 (90.8)	0.2436
≥7	4156	609 (14.7)	3547 (85.3)		420 (10.1)	3736 (89.9)	
Self-rated health							
Good	1448	96 (6.6)	1352 (93.4)	<0.0001	111 (7.7)	1337 (92.3)	0.0023
Poor	5337	818 (23.0)	4519 (77.0)		552 (10.3)	4785 (89.7)	
Sex							
Men	3024	468 (15.5)	2556 (84.5)	<0.0001	330 (11.0)	2691 (89.0)	0.0020
Women	3761	446 (11.9)	3315 (88.1)		333 (8.8)	3431 (91.2)	
Age							
Young-old	4615	567 (12.3)	4048 (87.7)	<0.0001	456 (9.9)	4159 (90.1)	0.6585
Old-old	2170	347 (16.0)	1823 (84.0)		207 (9.5)	1963 (90.5)	
Household income							
Quartile1	3107	435 (14.0)	2672 (86.0)	0.0739	281 (9.0)	2826 (91.0)	0.1254
Quartile2	1875	254 (13.6)	1621 (86.4)		207 (11.0)	1668 (89.0)	
Quartile3	1042	148 (14.2)	894 (85.8)		106 (10.2)	936 (89.8)	
Quartile4	728	76 (10.4)	652 (89.6)		67 (9.2)	661 (90.8)	
Missing	33						
Education level							
Under middle school	4932	678 (13.7)	4254 (86.3)	0.2408	440 (8.9)	4492 (91.1)	0.0001
High schooland above	1826	231 (12.7)	1595 (87.3)		220 (12.0)	1606 (88.0)	
Missing	27						
Marital status							
Yes	4565	637 (14.0)	3925 (86.0)	0.0964	456 (10.0)	4106 (90.0)	0.2831
No	2171	271 (12.5)	1900 (87.5)		199 (9.2)	1972 (90.8)	
Missing	52						
Physical activity							
Yes	2079	256 (12.3)	1823 (87.7)	0.0624	189 (9.1)	1890 (90.9)	0.2041
No	4660	652 (14.0)	4008 (86.0)		470 (10.1)	4190 (89.9)	
Missing	46						
Current smoking							
Yes	649	98 (15.1)	551 (84.9)	0.2110	47 (7.2)	602 (92.8)	0.0252
No	6052	807 (13.3)	5245 (86.7)		604 (10.0)	5448 (90.0)	
Missing	84						
High-risk alcohol drinking							
Yes	272	27 (9.9)	245 (90.1)	0.0777	20 (7.3)	252 (92.7)	0.1807
No	6435	879 (13.7)	5556 (86.3)		631 (9.8)	5804 (90.2)	
Missing	78						
Waist circumference							
Normal	3889	481 (12.4)	3408 (87.6)	0.0008	245 (9.0)	2508 (91.0)	0.0318
Obese	2753	419 (15.2)	2334 (84.8)		408 (10.5)	3481 (89.5)	
Missing	143						
Diabetes mellitus							
Yes	1501	268 (17.9)	1233 (82.1)	<0.0001	149 (9.9)	1352 (90.1)	0.5555
No	4495	516 (11.5)	3979 (88.5)		423 (9.4)	4072 (90.6)	
Missing	789						
Hypertension							
Yes	4247	660 (15.5)	3587 (84.5)	<0.0001	389 (9.2)	3858 (90.8)	0.0251
No	2530	253 (10.0)	2277 (90.0)		274 (10.8)	2256 (89.2)	
Missing	8						
Dyslipidemia							
Yes	2397	362 (15.1)	2035 (84.9)	0.0036	216 (9.1)	2167 (90.9)	0.1440
No	4388	552 (12.6)	3836 (87.4)		443 (10.2)	3913 (89.8)	

**Table 2:** The association of sedentary behavior and self-rated health with CVD or cancer.

	Cardiovascular Disease (CVD)		Cancer	
	OR	95% CI	OR	95% CI
Sedentary behavior				
0–6	1.00		1.00	
≥7	1.28	1.08–1.51	1.10	0.91–1.33
Self-rated health				
Good	1.00		1.00	
Poor	2.36	1.85–3.01	1.48	1.17–1.88
Sex				
Women	1.00		1.00	
Men	1.73	1.43–2.09	1.36	1.10–1.69
Age				
Young-old	1.00		1.00	
Old-old	1.37	1.15–1.63	1.00	0.81–1.23
Household income				
Quartile1	1.25	0.93–1.70	1.12	0.81–1.56
Quartile2	1.30	0.96–1.77	1.38	1.00–1.91
Quartile3	1.40	1.00–1.95	1.25	0.88–1.78
Quartile4	1.00		1.00	
Education level				
Under middle school	1.10	0.90–1.33	0.71	0.57–0.87
High school and above	1.00		1.00	
Marital status				
No	1.00		1.00	
Yes	1.21	0.92–1.36	0.94	0.75–1.17
Physical activity				
No	1.08	0.91–1.29	1.18	0.97–1.44
Yes	1.00		1.00	
Current smoking				
No	1.00		1.00	
Yes	0.98	0.75–1.28	0.63	0.45–0.90
High-risk alcohol drinking				
No	1.00		1.00	
Yes	0.52	0.33–0.84	0.64	0.37–1.10
Waist circumference				
Normal	1.00		1.00	
Obesity	1.40	1.41–1.92	0.88	0.73–1.19
Diabetes mellitus				
No	1.00		1.00	
Yes	1.41	1.19–1.67	1.06	0.86–1.30
Hypertension				
No	1.00		1.00	
Yes	1.45	1.21–1.73	0.94	0.78–1.14
Dyslipidemia				
No	1.00		1.00	
Yes	1.32	1.12–1.56	0.99	0.82–1.19

## Data Availability

All data files are available from the Korea Centers for Disease Control and Prevention database through the following URLs: https://knhanes.cdc.go.kr/knhanes/sub03/sub03_02_02.do. However, the process for data access and the user manual are only written in Korean.

## Supplementary Materials

The following are available online at https://www.mdpi.com/article/10.3390/ijerph18147426/s1, Table S1: Association of sedentary behavior and self-rated health with cardiovascular disease prevalence by sex, Table S2: Association of sedentary behavior and self-rated health with cancer prevalence by sex.
