# Supplementary material for: The Impact of Sedentary Behavior and Self-Rated Health on Cardiovascular Disease and Cancer among South Korean Elderly Persons Using the Korea National Health and Nutrition Examination Survey (KNHANES) 2014–2018 Data
Source: Int J Environ Res Public Health. 2021 Jul 12;18(14):7426. doi: 10.3390/ijerph18147426 (PMC8305062; doi:10.3390/ijerph18147426)
Supplement: Supplementary file 1 [file ijerph-18-07426-s001.zip › ijerph-1288005-supplementary.pdf]

Supplementary Materials

Supplementary Table S1. Association of sedentary behavior and self-rated health with cardiovascular disease by sex

	Total		Men		Women	
	OR	95% CI	OR	95% CI	OR	95% CI
Short SB and good SRH	1.00		1.00		1.00	
Short SB and poor SRH	1.91	1.32-2.78	2.36	1.46-3.83	1.49	0.83-2.68
Long SB and good SRH	1.03	0.65-1.59	1.33	0.76-2.34	0.61	0.28-1.31
Long SB and poor SRH	2.50	1.74-3.58	3.35	2.10-5.33	1.83	1.04-3.24

Adjusted for age, household income, education level, marital status, physical activity, current smoking, high-risk alcohol consumption, waist circumference, diabetes mellitus, hypertension, and dyslipidemia

SB, Sedentary behavior; SRH, Self-rated health

Supplementary Table S2. Association of sedentary behavior and self-rated health with cancer by sex

	Total		Men		Women	
	OR	95% CI	OR	95% CI	OR	95% CI
Short SB and good SRH	1.00		1.00		1.00	
Short SB and poor SRH	1.68	1.15-2.46	1.52	0.94-2.44	2.06	1.07-3.97
Long SB and good SRH	1.35	0.88-2.08	1.32	0.78-2.24	1.43	0.67-3.05
Long SB and poor SRH	1.75	1.21-2.53	1.72	1.09-2.72	2.01	1.06-3.81

Adjusted for age, household income, education level, marital status, physical activity, current smoking, high-risk alcohol waist circumference, diabetes mellitus, hypertension, and dyslipidemia.

SB, Sedentary behavior; SRH, Self-rated health
